# Targeting Epigenetic Regulators with HDAC and BET Inhibitors to Modulate Muscle Wasting

**DOI:** 10.3390/ijms242216404

**Published:** 2023-11-16

**Authors:** Lorenzo Nevi, Noora Pöllänen, Fabio Penna, Giuseppina Caretti

**Affiliations:** 1 Department of Biosciences, University of Milan, 20133 Milan, Italy; nevi.lorenzo@hsr.it; 2 Research Program for Clinical and Molecular Metabolism, Faculty of Medicine, University of Helsinki, 00014 Helsinki, Finland; 3 Department of Clinical and Biological Sciences, University of Torino, 10125 Torino, Italy

**Keywords:** epigenetics, muscle wasting, cachexia, sarcopenia, BET proteins, HDACs

## Abstract

Epigenetic changes contribute to the profound alteration in the transcriptional program associated with the onset and progression of muscle wasting in several pathological conditions. Although HDACs and their inhibitors have been extensively studied in the field of muscular dystrophies, the potential of epigenetic inhibitors has only been marginally explored in other disorders associated with muscle atrophy, such as in cancer cachexia and sarcopenia. BET inhibitors represent a novel class of recently developed epigenetic drugs that display beneficial effects in a variety of diseases beyond malignancies. Based on the preliminary in vitro and preclinical data, HDACs and BET proteins contribute to the pathogenesis of cancer cachexia and sarcopenia, modulating processes related to skeletal muscle mass maintenance and/or metabolism. Thus, epigenetic drugs targeting HDACs and BET proteins may emerge as promising strategies to reverse the catabolic phenotype associated with cachexia and sarcopenia. Further preclinical studies are warranted to delve deeper into the molecular mechanisms associated with the functions of HDACs and BET proteins in muscle atrophy and to establish whether their epigenetic inhibitors represent a prospective therapeutic avenue to alleviate muscle wasting.

## 1. Introduction

Muscle wasting occurs in different conditions and entails the progressive loss of skeletal muscle and function; this leads to loss of independence and negative effects on an individual’s quality of life. Muscle loss can be induced by multiple causes such as immobility [1], malnutrition/starvation [2], pharmacological treatment [3], or a wide range of diseases involving the muscle tissue, for instance in cachexia secondary to cancer, renal failure, and chronic obstructive pulmonary diseases [4,5]. Likewise, aging is accompanied by a progressive decline in muscle mass, quality, and strength, which can become a pathological state known as sarcopenia that affects more than half of individuals aged >80 years [6]. In addition, loss of muscle mass and function develops in patients affected by genetic muscular dystrophies, such as Duchenne muscular dystrophy (DMD), facioscapulohumeral muscular dystrophy (FSHD), and limb–girdle muscular dystrophy (LGMD) [7].

Although the onset and the underlying molecular mechanisms driving muscle wasting differ in such pathological states, muscle loss consistently represents a negative prognostic factor for the progression of the disorder [4].

Epigenetic factors influence chromatin structure and gene expression in all tissues throughout life and undergo many key modifications during disease onset and aging. Several chromatin factors have been described to interplay with transcription factors to modify the epigenetic landscape at specific loci in muscle diseases. In addition, since epigenetic changes are reversible and can be modified by pharmacological intervention, epigenetic drugs may become a pivotal tool to temper the progression of muscle atrophy. Among the multiple classes of epigenetic inhibitors currently on the market, histone deacetylase (HDAC) inhibitors have received the broadest attention because of their employment in the clinics as a pharmacological avenue for cancer treatment [8,9].

HDAC inhibitors have been extensively studied in muscle dystrophy mouse models, [10] and the givinostat molecule was approved in 2023 for Duchenne muscular dystrophy [9,11,12]. HDAC’s role in skeletal muscle and in the mononucleated cells within the muscle tissue, as well as the impact of HDAC inhibitors in muscular dystrophies, has been recently accurately reviewed by Sandonà et al. [13] and will not be discussed in this review.

More recently, a novel class of epigenetic inhibitors has been rapidly developed targeting the bromodomain and extraterminal domain (BET) family of proteins, and the first efforts have been made to test their impact on skeletal muscle wasting [14,15,16].

In this review, we will focus our attention on the molecular mechanisms involving HDACs and BET proteins in sarcopenia and cancer cachexia and how epigenetic modulators targeting these two classes of epigenetic factors may contribute to alleviating muscle wasting in the abovementioned pathologies.

## 2. Signaling Pathways Involved in Sarcopenia and Cachexia

**Figure 1:**
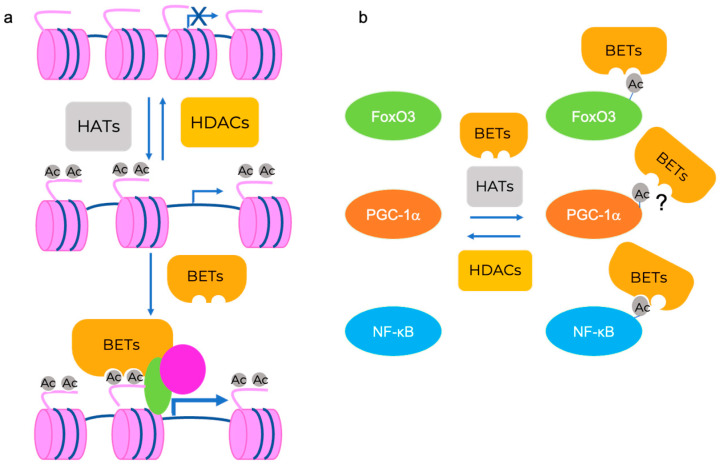
HDAC and BET proteins may contribute to muscle wasting through chromatin and non-histone protein modifications. (**a**) Transcription is regulated by the interplay between the HAT writer and HDAC erasers. Histone acetylation promotes transcription through the association of several different proteins and complexes, including the BET readers. (**b**) Transcription factors and co-activators are HATs and HDACs substrates. The association of the acetylated form of Foxo3 and NF-κB with BRD4 has been described in other contexts [18,19] and may play a role in skeletal muscle also.

**Figure 2:**
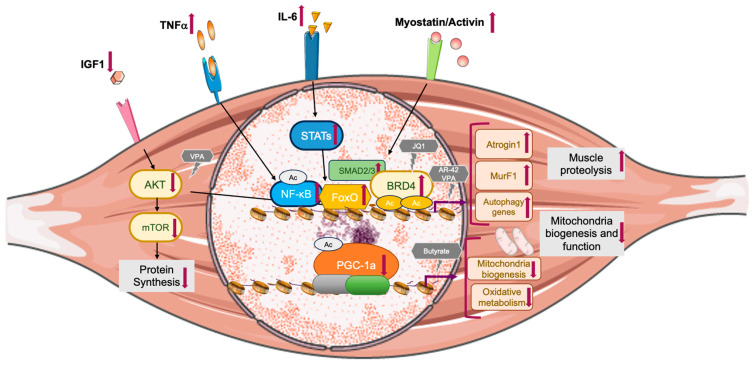
The signaling pathways contributing to cancer cachexia and sarcopenia are highlighted, focusing on downstream nuclear targets and the transcriptional programs affected by extracellular factors that lead to muscle wasting. The grey boxes depict the intracellular processes primarily involved in sarcopenia and cachexia, whereas the dark grey hexagons represent epigenetic drugs affecting the underlying molecular processes. Ac represents acetylated lysine residues. Image created with PowerPoint and Smart Servier.

Chromatin factors can contribute to muscle wasting in two ways: (i) by directly modulating histone modifications at regulatory regions relevant for transcriptional regulation of pro-atrophy and metabolic genes or (ii) by modulating the activity of transcription factors and co-activators through their post-translational modifications (Figure 1 and Figure 2). To provide a few examples, we will first describe the signaling pathways that drive muscle wasting in sarcopenia and cachexia. Then, we will define how HDAC and BET epigenetic factors and their pharmacological modulators impact muscle wasting.

Sarcopenia is a complex condition and its onset and progression have been proposed to depend on multiple factors, such as changes in hormones, immobility, age-related muscle changes, nutrition, neuromuscular factors, oxidative stress, and inflammation [17]. Furthermore, these factors often interact with one another, making the development and progression of sarcopenia a multifaceted process. At the intracellular level, these factors affect key processes in skeletal muscle, such as proteostasis, mitochondria homeostasis and muscle regeneration.

Cancer-associated cachexia is a complex metabolic syndrome that accompanies various malignancies and is associated with increased morbidity and mortality. Muscle wasting in cachexia is mainly caused by the loss of proteostasis, implying impaired protein synthesis and increased proteolysis, and is primarily driven by the ubiquitin–proteasome system (UPS), autophagy, and the calpain pathway. The primary mediators of cancer cachexia are inflammatory signals, the TGFβ family members myostatin and activin A, hormones, and a reduction in anabolic signals.

Thus, cachexia and sarcopenia share an unbalanced equilibrium between protein degradation and protein synthesis; however, these events are characterized by distinct underlying molecular mechanisms and causes in the two pathologies. Although there are similarities in some of the molecular mechanisms involved in both cachexia and sarcopenia (e.g., inflammation and altered muscle proteostasis), the primary drivers and the severity of these mechanisms differ between the two conditions [4].

### 2.1. Inflammatory Signaling Pathways

Pro-inflammatory cytokines such as tumor necrosis factor alpha (TNF-α), interleukin 6 (IL-6), and interleukin 1 (IL-1) are considered as the primary mediators of cancer-associated cachexia, since the multiple inflammatory cytokines originating from the tumor or stromal cells contribute to the activation of muscle proteolysis [5]. Low-grade chronic inflammation, often referred to as “inflammaging”, also plays a role in sarcopenia, although its impact is less severe than the inflammation seen in cachexia [20]. In chronic inflammation, IL-6 secretion leads to the generation of a damaging environment for the muscle cells. In fact, a strong association between high IL-6 levels and loss of body mass has been observed in the elderly [21]. An observational study with more than 2000 elderly people revealed that higher TNF-α levels were associated with decreased muscle mass and strength [22]. In addition, during aging, senescent cells express the senescence-associated secretory phenotype (SASP) as a paracrine signaling pathway. “Inflammaging” and SASP production in senescent skeletal muscle cells converge on the activation of nuclear factor kappa B (NF-κB) signaling (Figure 2).

### 2.2. Protein Degradation

The two main protein degradation systems, the ubiquitin–proteosome system and autophagy, are highly activated in cachexia [23,24,25], causing loss of muscle mass and strength. Pro-inflammatory cytokines induce the activation of the signal transducer of activation of transcription 3 (STAT3), NF-κB, and forkhead box 1/3 (FoxO1/3) transcription factors, which promote the transcription of the muscle-specific E3 ubiquitin ligases Atrogin 1 and MuRF1 and other atrogenes, which promote UPS activation [26,27,28] (Figure 2).

Although the initial reports suggested an increase in muscle proteasome activity with aging in rats, these data do not incorporate the robust activation program of the atrogenes that is typically associated with the rapid muscle atrophy observed in cachexia [29]. The role of MuRF1 and Atrogin 1 in sarcopenia has been controversial: certain reports show a modest increase in the expression of these E3 ubiquitin ligases with aging in the mouse TA [30], others show no change in humans [31], and others a reduction in rats [32]. The inconsistent role of muscle-specific E3 ubiquitin ligases has been further supported by the finding that the proteolytic systems do not significantly increase in the elderly and in mouse models [33].

Autophagy also increases in cachexia, where the transcription factors FoxO1/3 activate autophagy-related genes such as *MAP1LC3a* and BNIP3, encoding for the LC3 and BNIP3 proteins respectively [25].

Conversely, in sarcopenic muscles, a defective formation of the autophagosome and an impairment of the autophagic flux has been observed. This is probably due to a downregulation of the LC3 gene and protein expression pattern (LC3 is involved in the autophagosome formation) and LAMP-2 (a crucial protein that mediates fusion of the autophagosome with the lysosome) [34,35]. Consequently, sarcopenia is characterized by an insufficient clearance of intracellular waste material and to the accumulation of protein aggregates and damaged mitochondria that are not removed, eventually leading to the blockade of protein synthesis, thus causing muscle loss. Indeed, dysregulated elimination of mitochondria through mitophagy has been suggested as a driver of sarcopenia [36].

### 2.3. Protein Synthesis and the IGF1/AKT/mTOR Pathway

The anabolic growth factor insulin-like growth factor 1 (IGF1), Akt, and mammalian target of rapamycin (mTOR) signaling pathways play a central role in modulating skeletal muscle protein turnover in physiological and pathological conditions [25,33]. During the catabolic state observed in cachexia, the reduced activation of the IGF1/AKT pathway results in reduced AKT-dependent FoxO inhibition and in the activation of pro-atrophic transcriptional programs that are under the control of FoxO1/3 (Figure 2). Several in vivo and in vitro studies [25,26,37,38] have demonstrated that FoxO1 and FoxO3 play a primary role in muscle atrophy, actively promoting the expression of E3 ubiquitin ligases and of autophagy genes in cancer cachexia. Likewise, in the aging muscle, protein synthesis is defective. In humans, rats and mice, sarcopenia is characterized by the reduced sensitivity of muscle protein synthesis to the anabolic signals triggered by amino acids, insulin, and various nutrients and hormones associated with protein metabolism after food intake [33,39,40,41,42]. Several atrophy-related genes are under the control of transcription factors such as FoxO3 and NF-κB [33] and various post-translational modifications control FoxO1 and FoxO3 activity, including activation through phosphorylation by MST1 or AMPK [43] and inactivation through phosphorylation by Akt [33], deacetylation by sirtuin 1 (Sirt1) [44], or by binding with the oncogene JUNB or the PPARG coactivator 1 alpha PGC-1α [4].

### 2.4. Mitochondrial Abnormalities and the Sirt1/PGC-1α Pathway

**Figure 3:**
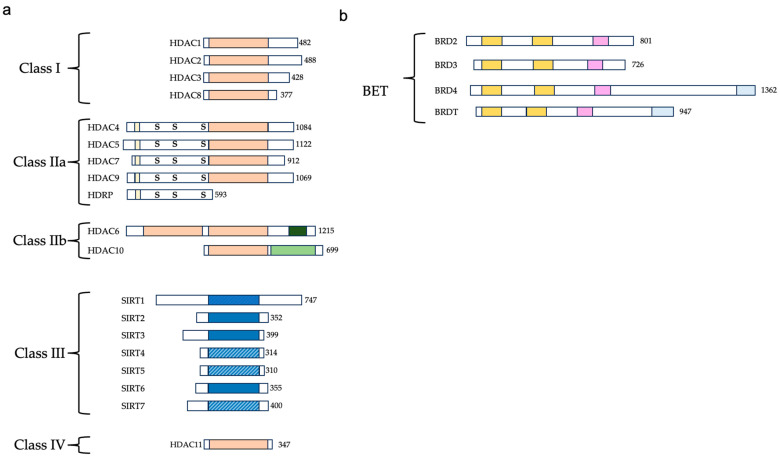
HDAC and BET family member classification and domain composition. (**a**) HDAC family: on the left, we indicate in light orange the conserved deacetylase domain of the classical HDACs and in blue the catalytic domain of the sirtuins. The solid blue color indicates deacetylase activity and the striped pattern indicates domains in which the deacetylase activity remains elusive or is associated with other catalytic activities. We reported in light yellow myocyte-specific enhancer factor 2A (MEF2)-binding site, the S symbol indicates the key serine residues that are phosphorylated, in green are the leucine-rich regions, and in dark pink we indicate C-terminal zinc finger (ZnF). (**b**) BET family members: on the right, we indicate in yellow the two bromodomains, in pink the extra-terminal domain (ET), and in light blue the C-terminal domain (CTD).

Aging is characterized by mitochondrial dysfunction in the muscles of both humans and rodents. This dysfunction includes impaired oxidative phosphorylation, reduced mitochondrial DNA content and accumulation of mutated mitochondria DNA, dysfunctional fission/fusion, and impaired mitophagy [45,46,47,48]. The AMPK–SIRT1–PGC-1α signaling pathway plays a critical role in sensing energy levels and subsequently regulating mitochondrial production, energy metabolism, and managing oxidative stress [49]. AMPK orchestrates the transition between anabolic and catabolic metabolism [50]. In muscle, this transition is achieved through the AMPK-dependent activation of an additional metabolic sensor, SIRT1, which leads to the deacetylation of downstream targets such as PGC-1α [51] (Figure 3b). Thus, whereas in the healthy muscle the AMPK–SIRT1–PGC-1α axis plays a pivotal role in regulating mitochondrial biosynthesis and homeostasis, in the aging mouse muscle, PGC-1 α acetylation was reported to increase compared with the young muscle, hinting at a much reduced ability to transactivate its target genes [52] (Figure 2).

A recent study focused on the transcription profile of muscles of sarcopenic individuals and age-matched controls revealed that individuals with sarcopenia consistently exhibited significant changes in the expression of genes related to mitochondrial bioenergetic dysfunctions. These changes included a reduction in the signature genes regulated by PGC-1α and downregulation of genes associated with oxidative phosphorylation and mitochondrial proteostasis. Functionally, these transcriptional changes resulted in fewer mitochondria, reduced expression and activity of mitochondrial respiratory complexes, and low levels of NAD+ in sarcopenic muscles [53]. Furthermore, it has been shown that the decreased bioenergetic availability in skeletal muscle is associated with a reduction in the number of mitochondria and their functionality [54].

In experimental models of cancer cachexia, muscle is characterized by imbalanced mitochondrial dynamics (increased fission and reduced fusion), decreased activity of respiratory chain complexes, and mitophagy [55,56].

A genome-wide analysis of a cancer-induced cachexia mouse model revealed reduced expression of the genes related to mitochondrial fusion, fission, ATP production, and mitochondrial density, along with increased expression of genes involved in detoxifying reactive oxygen species (ROS) and mitophagy [57].

Therefore, it appears that imbalances in mitochondrial dynamics, mitophagy, and oxidative activity are the key contributing factors for cachexia. Of note, although downregulation of PGC-1α levels was reported in rats bearing AH-130 ascites tumors [58], PGC-1α levels were found to be unchanged in other cancer cachexia models [59,60,61,62]. Despite these inconsistent data, further information related to PGC-1α activation through AMPK-mediated phosphorylation and SIRT1 deacetylation are lacking in these experimental models.

### 2.5. Myostatin/Activin A Pathway

Another pathway involved in protein hypercatabolism associated with muscle wasting is the myostatin/activin A pathway. Myostatin and activin A are transforming growth factor-beta (TGF-β) superfamily ligands that recognize two related transmembrane type I and type II serine/threonine kinase receptors to activate downstream signal transduction. Activation of the myostatin/activin A signaling pathway results in SMAD2 and SMAD3 phosphorylation [63], which regulates transcriptional responses leading to atrogene activation in cancer cachexia [64,65,66,67] (Figure 2). Serum myostatin levels were inconsistently reported to be unchanged [68,69], increased [70], or reduced in sarcopenic versus young individuals [71]. Nevertheless, when a myostatin antagonist was administered to elderly mice, it resulted in an improvement in muscle regeneration during the onset of sarcopenia [72].

### 2.6. Decreased Regenerative Capacity

Satellite cell function and abundancy significantly decline in sarcopenic conditions, and the progressive loss of regenerative capacity strongly impairs the function of the old skeletal muscle [73]. In cancer cachexia, muscle stem cells isolated from C26 tumor cell-bearing mice maintain the ability to proliferate and differentiate in vitro, but both their proliferation and differentiation potential were compromised in vivo [74]. These findings suggest that, in cancer cachexia, the muscle microenvironment affects and compromises the regenerative cues associated with healthy regeneration [75].

## 3. HDACs and Their Inhibitors

Histone post-translational modifications play a key role in gene transcription, shaping chromatin structures, and nuclear organization. One of the most relevant epigenetic signaling mechanisms in the differentiated tissue is ascribed to the balanced interplay between histone acetyltransferases (HATs) and HDACs, which tightly regulates the histone acetylation levels at regulatory regions (Figure 1a). In this context, HDACs remove acetyl groups from the lysines at the N-terminal tail of histones and promote a state of histone hypoacetylation that, in turn, relaxes the chromatin structure, ultimately hampering the progression of the transcriptional process. HATs and HDACs also target acetylated non-histone proteins, such as transcription factors, structural proteins, and enzymes, modulating their function in a different context [76]. Although HATs and HDACs belong to the class of chromatin writers, chromatin readers recognize acetylated lysines within histones or regulatory factors through protein modules, such as the bromodomains (BDs) (Figure 1b) [77]. Because of the key function of acetylation in gene transcription, HDACs and BD-containing proteins modulate a plethora of processes in physiological and pathological conditions, including development, cell growth and differentiation, and cancer [10].

In mammals, eighteen HDACs have been described that exploit two distinct catalytic mechanisms: 11 rely on zinc as a cofactor for their function and promote the hydrolysis of amide bonds using water as a nucleophile (HDAC1-11). In addition, seven sirtuins (1–7) utilize NAD+ as a cofactor to transfer the acyl group to the ribose sugar at the C2 position. These two families are further divided into four classes: class I (HDAC1-3 and HDAC8), class IIa (HDAC4, HDAC5, HDAC7, and HDAC9), class IIb (HDAC6 and HDAC10), class III (sirtuin 1–7), and class IV (HDAC11) [78] (Figure 3a).

HDAC inhibitors are formed by a cap group, a linker part, and a Zn-binding group; they are further divided into two structural classes based on the zinc binding group: hydroxamic acids and aminoanilides. Examples of the hydroxamic-acid-type HDAC inhibitors are vorinostat (also known as suberoylanilide hydroxamic acid, SAHA) [79], belinostat (PXD101), givinostat, AR-42, and panobinostat (LBH589). In 2006, vorinostat became the first HDAC inhibitor to obtain FDA approval for the treatment of refractory primary cutaneous T-cell lymphoma [80].

Benzamides represent another category of synthetic HDAC inhibitors characterized by their bidentate coordination of the carbonyl oxygen and aniline nitrogen with the active site zinc cation. These compounds exhibit weaker metal binding affinity compared with hydroxamic acids and display distinctive kinetic behavior, including slow and tight binding to HDACs [8]. Clinical candidates from this group of synthetic HDAC inhibitors include tacedinaline, etinostat, mocetinostat, tucidinostat, domatinostat, and CXD [8].

Among the benzamides, tucidinostat (chidamide) is the only approved compound; it is authorized for use in treating patients with recurrent or refractory peripheral T-cell lymphoma [81].

In addition, short-chain fatty acid inhibitors include valproic acid and butyrate, whereas cyclic peptide inhibitors include the class I inhibitor romidepsin [82].

Additionally, several selective HDAC6 inhibitors, such as CKD-504, CKD-506, CS3003, HG116, and KA2507 are currently in phase I clinical trials [83] (Table 1).

## 4. BET Proteins and Their Inhibitors

BDs are highly conserved protein interaction modules with a critical role in recognizing ε-N-lysine acetylation motifs. The BETfamily of proteins is distinguished by the presence of two consecutive bromodomains and an additional terminal domain. The mammalian BET family encompasses BRDT, BRD2, BRD3, and BRD4 [84] (Figure 3b). These proteins possess the unique ability to specifically detect and bind to acetylated lysine residues, thereby influencing gene transcription. The most studied BET family member is BRD4, which is ubiquitously present in all tissues and has been widely studied in the cancer field because of its pivotal role in oncogene expression regulation through super-enhancer (SE) organization [85]. In addition to regulating transcription initiation through the association with transcription factors and chromatin regulators, BRD4 also interacts with transcription elongation complexes (P-TEFb) to govern the transcription of signal-inducible genes. It engages with P-TEFb, stimulating its kinase activity to phosphorylate RNA polymerase II and facilitating the resumption of transcription [86]. Of relevance to non-cancer related diseases, BRD4 plays a key role in the inflammatory response in sepsis and other conditions [87,88].

BET proteins have obtained increased attention in biomedical studies due to their exceptional susceptibility to potent and highly targeted inhibitors, making them an attractive target for drug development [89]. JQ1 is a thieno-triazolo-1,4-diazepin and the most extensively studied BET inhibitor [90,91,92,93]. BRD4, along with other members of the BET family, possesses an acetyl-lysine binding site capable of binding the acetylated lysines found on histone tails or proteins [94]. JQ1 can directly bind to this acetylated lysine binding site, fully occupying the entire binding pocket [94]. This binding is further stabilized through hydrophobic interactions with conserved BET residues. Intriguingly, in cell lines and animal models JQ1+ not only prevents BRD4 from interacting with acetylated residues, thus inhibiting its activity, but also displaces it from the chromatin [15,16,85,92]. JQ1 cannot be employed in clinical trials because of its poor pharmacokinetic profile and low oral bioavailability [95]. Thus, JQ1 analogs and derivatives have been developed that show better tolerance and minor toxicity [96], such as PFI-1, I-BET762, and OTX015 [97]. Although these human-suitable inhibitors display a better pharmacokinetic profile, they still present side effects in humans that restrict the dose escalation and, in some cases, lead to treatment discontinuation [98]. A second generation of BET inhibitors has been designed to reduce dose-limiting toxicity by selectively targeting one of the two BDs. Among this class, RVX-208 mainly blocks BD2 function [99], whereas GSK778 is a BD1 selective inhibitor [99]. CPI-0610 is another second-generation BET inhibitor with a molecular structure similar to JQ1. CPI-0610 is currently in phase 3 clinical trials for myelofibrosis (NCT04603495) and displays higher potency and selectivity and reduced toxicity than the first-generation compounds [100,101,102].

Importantly, whereas the BD1 and BD2 second-generation inhibitors are effective in blocking cell proliferation and cell survival in cancer models, BD2-selective inhibitors are largely ineffective [99]. Conversely, BD2-selective inhibitors ameliorate inflammatory diseases in preclinical models [99] and are good candidates for inflammation-induced cardiac dysfunction and SARS-CoV-2 infection [103] (Table 1).

## 5. How Are HDACs and BET Proteins Implicated in Sarcopenia and Cachexia?

HDACs have been shown to directly participate in the regulation of different transcriptional programs in the wasting skeletal muscle, such as in the transcriptional regulation of inflammation and oxidative stress genes, muscle regeneration, protein homeostasis, and mitochondrial function [104]. Thus, dysregulation of HDAC activity might exacerbate these processes, thereby accelerating muscle loss. Concurrently, HDAC pharmacological modulation may be beneficial in the attenuation of muscle loss.

In addition to histones, HDACs target non-histone proteins, e.g., transcription factors (Figure 1b). In the context of muscle mass regulation, acetylation of specific lysine residues can impact the cellular localization (activation/inactivation) of transcription factors such as FoxO1/3, signal transducer and activator of transcription (STAT)-1 and STAT3, NF-κB, and the coactivator PGC-1α. For instance, Sirt1 blocks NF-κB activity by deacetylating the p65 subunit and preventing FoxO1/3 activation, thus reducing *Atrogin 1* and *MuRF1* transcription [44]. PGC-1α, following AMPK dependent phosphorylation, is deacetylated by Sirt1, and this results in an increased nuclear localization and activation of its target genes [105].

BET proteins were shown to interact directly with acetylated transcription factors, such as the p65 subunit of NF-κB [19] and FoxO3 [18]; however, these interactions were not proven in skeletal muscle, nor were they reported to play a role in muscle wasting.

### 5.1. HDAC and BET Proteins Regulate Skeletal Muscle Mass Maintenance

HDACs, and to a lesser extent BET proteins, have been reported to play a role in the regulation of muscle mass, either by regulating the expression of *Atrogin 1*, *MuRF1*, and autophagy genes or modulating other catabolic signals, for instance the myostatin pathway.

HDAC1, 4, 5, 6, and SIRT1 have been proposed to play a critical role in regulating the onset and progression of skeletal muscle atrophy. For example, mRNA expression of *HDAC2*, *HDAC4*, *HDAC6*, and *SIRT1* is increased in the skeletal muscle of animals subjected to nutrient deprivation, denervation, or cast immobilization [106]. Only cast immobilization and denervation were associated with increased mRNA levels of *HDAC1* and *HDAC3*, whereas decreased levels of *HDAC7* and *HDAC9* were observed in different models of muscle atrophy [106].

HDAC1 favors FoxO activation during nutrient deprivation, and TSA treatment prevents muscle atrophy following starvation [106]. HDAC4, 5, and 7 recruit HDAC3, thereby causing deacetylation and activation of FoxO1/3 transcription factors and the subsequent transcriptional induction of pro-atrophy genes [107,108]. In addition, in myoblast cell lines and animal models, HDAC6 activates FoxO3a under the conditions of denervation muscle atrophy [109].

Overexpression of HDAC4 has been shown to reduce myofiber cross-sectional area, whereas its deletion improves the phenotype in denervated mouse muscles [110]. Similarly, HDAC6 is overexpressed during muscle wasting and its inactivation has been shown to protect against denervation-induced muscle atrophy [109]. HDACs have also been proposed to regulate the expression of atrogenes, such as the muscle-specific ubiquitin ligases *MuRF1* and *Atrogin 1/MAFbx*, by acting on two relevant transcription factors, myogenin and the FoxOs. In denervated muscles, HDAC4 was shown to interact with FoxO3 and to inhibit its degradation, thus enhancing FoxO3 signaling on its targets [111]. Acetylated FoxO3 is translocated from the nucleus to the cytoplasm and subsequently degraded; however, deacetylation increases transcriptional activity. Myogenin, an important muscle regulatory factor in late myogenesis, also participates in the induction of *Atrogin 1/MAFbx* during denervation, a process requiring HDAC4 [110].

Although the molecular mechanisms involving HDACs, FoxO3, Atrogin 1/MAFbx, and MuRF1 have not been specifically investigated in the context of cancer cachexia; they may fine tune the FoxO3 activity in skeletal muscle during cancer cachexia as well.

In old rats, HDAC4 levels are mildly decreased in gastrocnemius with aging but are significantly upregulated in a model of disuse atrophy [112]. In normal conditions, HDCA5 modulates TFEB-mediated *MuRF1* transcriptional activation in mouse skeletal muscle, whereas angiotensin II promotes muscle atrophy favoring HDAC5 cytoplasmic translocation and *MuRF1* expression through TFEB. This pathway may play a role in angiotensin-II-induced muscle atrophy in congestive heart failure patients [113].

The class I and II HDAC inhibitor TSA prevents the increase in *MuRF1* expression in the soleus in a model of unloading-induced muscle atrophy [114].

TSA also promotes an induction of follistatin, an antagonist of myostatin, that promotes an increase in muscle mass and strength [115]. Myostatin, MurRF1, and Atrogin 1 levels are also reduced by BET inhibition through the small molecule JQ1 in C2C12 myotubes in an in vitro model of dexamethasone-induced atrophy [116].

Sirt1 has been linked to improved metabolic function in the aging skeletal muscle. For instance, Sirt1 stimulates the activity of PGC-1α through deacetylation [117] and thus protects against sarcopenia. Additionally, supplementation with an NAD+ precursor as a Sirt1 cofactor has demonstrated protective effects against age-related metabolic alterations in the skeletal muscle of animal models in a Sirt1-dependent manner [118]. Sirt1 also promotes FoxO1 and FoxO3a deacetylation in vivo and in vitro and decreases FoxO protein activity in fasted mice [44]. On the other hand, FoxO1 and FoxO3a are activated by HDAC1, which induces the muscle atrophy associated with muscle disuse [106]. Sirt1 also negatively modulates the transcriptional activation mediated by the NF-κB transcription factor by deacetylating the lysine 310 of the p65 subunit, thus reducing its transcriptional ability. This axis has been proposed to play a role in cancer cachexia through the regulation of the Nox4 subunit and oxidative stress [119]. However, in the muscle of old rats, a TNFα increase does not upregulate p65 protein levels or NF-κB’s DNA-binding ability [120].

Despite this line of evidence suggesting multiple regulatory roles for HDACs in muscle atrophy, knowledge is lacking regarding their specific functions and the underlying molecular mechanisms in sarcopenia and cachexia.

In the C26 experimental model of cancer cachexia, oral administration of the class I (HDAC1, 2, 3, and 8) and IIb (HDAC6 and 8) AR-42 inhibitor extended survival significantly, while preventing the loss of muscle and fat tissue mass, preserving muscle fiber size, and enhancing muscle strength. Additionally, AR-42 hindered the upregulation of MuRF1 and atrogin-1 mRNA levels. Although AR-42 treatment did not affect serum TNFα levels in C26-tumor-bearing mice, it reduced levels of serum IL-6 and intramuscular IL-6Rα mRNA expression [121] (Figure 2). This anti-cachectic effect was reproduced in the Lewis lung cancer cachexia model but, notably, it was not observed following administration of the class I, II, and IV inhibitor vorinostat or the class I inhibitor romidepsin [121], as well as of the class I and IIa inhibitor valproic acid (VPA) and the class I, II, and IV inhibitor TSA [122,123]. These findings suggest that AR-42’s effects are mediated by specific interactions with multiple transcriptional regulators or, alternatively, this compound may specifically block HDAC activity in certain regulatory complexes containing HDACs. Further investigations are warranted to better characterize the molecular mechanism underlying muscle preservation following AR-42 treatment in tumor-bearing mice.

A more recent study reported that higher doses of valproic acid ameliorates muscle wasting in the C26 and LLC experimental models. In fact, in the C26 and LLC experimental models of cancer cachexia, VPA treatment preserved the muscle mass and cross-sectional area in TA muscles. Following VPA treatment, muscles displayed reduced levels of CCAAT/enhancer binding protein beta (C/EBPb), which is necessary for Atrogin 1 upregulation in tumor-bearing mice. Interestingly, VPA does not have any impact on FoxO activity in this model. Overall, VPA treatment also improved pAkt/Akt and p-S6/S6 ratio, suggesting that it enhances protein synthesis and attenuates protein catabolism in muscles from tumor-bearing mice [124] (Figure 2).

The impact of HDACs and their inhibitors in the aging muscle has not been extensively studied. One report shows that mice fed with a butyrate-containing diet for 10 months, starting at 16 months of age, exhibited increased mitochondrial biogenesis in the skeletal muscle of old mice and a reduction in the markers of oxidative stress. The increased muscle mass was not due to a reduction in E3-ligase-mediated proteasomal degradation [125] (Figure 2).

Atrogenesexpression was shown to also be dependent on BRD4 and BRD2 in muscle from C26-tumor-bearing mice. Administration of the BET inhibitor JQ1 in this experimental model of cancer cachexia prevented weight loss and spared muscle and adipose tissues, extending survival without impacting directly on tumor growth. At the onset of cancer cachexia, the increased FoxO3 recruitment and histone acetylation facilitated the association of BRD4 at pro-atrophy genes, promoting their RNA-Pol-II-mediated transcription. Increased FoxO3 occupancy at pro-atrophic genes such as *MuRF1*, *MAFbx/atrogin-1*, and *GABARAPL1* is facilitated by AMPK-mediated FoxO3 phosphorylation, which in turn is induced by elevated systemic IL-6 levels and AMPK activation in skeletal muscle (Figure 2). Additionally, BRD4 and BRD2 are directly involved in the transcriptional regulation of *IL-6* and the parathyroid-hormone-related protein *PTHrP* in C26 tumors. Following JQ1 treatment, the expression of *IL-6* and *PTHrP* in C26-cell-derived tumors is diminished, indicating that JQ1 also alters the tumor transcriptional program, preventing the upregulation of pro-cachectic factors without affecting tumor mass [15].

JQ1 treatment in aged mice reduced the extracellular matrix upregulation in skeletal muscle and fibrogenic conversion of satellite cells, also restoring their myogenic differentiation potential [126]. In addition, BRD4 was shown to occupy the chromatin regulatory regions of NADPH oxidase subunits in the mdx muscle. This increased association led to elevated levels of reactive oxygen species (ROS), inflammation, fibrosis, and necrosis [16]. The administration of JQ1 has been shown to effectively reduce NADPH subunit transcript levels and ameliorate the phenotype of mdx mice [16].

Because of the key roles played by oxidative stress, inflammation, and fibrosis in sarcopenia, pharmacological blockade of BET proteins may also target these processes when they are involved in the aging skeletal muscle.

Overall, this line of evidence suggests that further preclinical studies on the impact of multiple HDAC and BET inhibitors are necessary to better dissect the impact of these drugs on the progression of cancer cachexia and sarcopenia. For instance, little is known about the role of HDAC and BET inhibitor administration on neuromuscular junctions and the decline of motor neurons or on the mitochondrial dysfunctions observed in sarcopenia.

### 5.2. Epigenetics—Metabolism Crosstalk in Cachexia and Sarcopenia

Muscle wasting with disrupted metabolic homeostasis is a hallmark of cachexia and sarcopenia. Chronic exposure to factors causing muscle exhaustion, such as inflammatory factors and mitochondrial dysfunction in disease-induced cachexia and aging-related sarcopenia, results in metabolic reprogramming. This process is closely intertwined with epigenetics [127], which connects environmental factors and genetics in an individual’s phenotype. However, the crosstalk between epigenetics and metabolism in muscle wasting remains partly unclear, especially regarding causality, disease trajectory, and therapy perspectives.

The mechanisms underlying the disturbed metabolism in muscle atrophy include either anabolic resistance with diminished protein synthesis in sarcopenia or accelerated protein degradation in cachexia [128]. Other metabolic alterations that may occur, especially in cancer cachexia, are accelerated glucose production following excess lactate secretion by the tumor, which supports tumor growth, and increased lipolysis triggered by inflammatory factors [129]. These adaptations modify the cellular concentrations and fluxes of intermediate metabolites that are utilized as substrates and cofactors in subsequent epigenetic modifications, generating a bi-directional crosstalk between metabolic adaptation and epigenetics. The most relevant metabolites in this crosstalk include acetyl-CoA, nicotinamide adenine dinucleotide (NAD+), α-ketoglutarate, and S-adenosylmethionine (SAM). Acetyl-CoA and NAD+ play crucial roles in regulating the acetylation status of histones. Acetyl-CoA is at the center of glucose and lipid metabolism and its concentration reflects the cellular metabolic state. It maintains cell’s acetylation capacity by donating acetyl groups for histones in a reaction catalyzed by histone acetyltransferases (HATs) [130]. Similarly, NAD+ operates at the core of the energy conversion processes in the TCA cycle and OXPHOS but also functions as a substrate for sirtuins (SIRTs). The equilibrium between the HAT and HDAC activities is responsible for the conformational changes in chromatin with transcription activation (chromatin open) and inactivation (chromatin closed) capabilities, respectively. NAD+ regulates mitochondrial metabolism and promotes sirtuin activity, whereas muscle NAD+ depletion has been reported in cachectic mice and sarcopenic humans [53,131]. Therefore, NAD+ loss in muscle atrophy may result in sirtuin deactivation and pronounced histone acetylation, alongside a disturbed gene expression profile. We recently showed that NAD+ precursor therapies, on the other hand, are promising approaches for muscle wasting conditions [131], although a clear demonstration of the contribution of NAD+ repletion to the epigenetic control of muscle metabolism is lacking and the scant data available only allow speculation on potential mechanisms. For instance, in cachectic mice, NAD+ repletion with niacin slightly increased the mRNA expression of the mitochondrial Sirt3 but the enzymatic activity of sirtuin was not determined. Similarly, some indications of changes in DNA methylation (discussed below) upon nicotinamide riboside were reported in a population of mostly obese individuals [132], supporting the concept that the NAD+ status takes part in the control of muscle epigenetic regulatory processes.

One-carbon metabolism provides another metabolic regulatory point for epigenetic modifications. It connects metabolism of amino acids, specifically methionine, serine and glycine, to the methionine cycle to provide methyl groups for post-transcriptional modifications [133]. These amino acids are utilized to produce SAM. With the help of DNA methyltransferases (DNMTs), SAM provides a methyl group for DNA regions with CpG sites, which usually prevent the binding of transcription factors and other proteins, leading to repressed gene expression. Conversely, DNA demethylation occurs via the TCA cycle intermediate α-ketoglutarate and is mediated dioxygenases [134]. Few reports have been published regarding methylation in muscle atrophy and especially cachexia. In sarcopenic men, muscle methylome was significantly altered, especially in genes participating in oxidative phosphorylation and myogenesis [135]. Furthermore, inhibition of enhancer of zeste homologue 2 (EZH2), a histone methyltransferase and one of the differentially expressed targets in sarcopenia, impacted the methylation of OXPHOS-related genes and improved mitochondrial respiration in human primary myoblasts [135].

If the causal relationship between the metabolic alterations and the epigenome status still requires some solid demonstrations, a strong line of evidence exists linking the modulation of epigenetics and the consequent impact on muscle metabolism. Indeed, HDACs provide a therapeutic target for sarcopenia and cachexia, given that many HDACs participate in the regulation of muscle mass and metabolism in wasting conditions [136]. The inhibition of HDAC activity has resulted in improvements, for example, with AR-42 in C26-tumor-bearing mice [137] and with entinostat in cardiac complications in rodent cachexia [138]. In addition to the control of processes regulating muscle atrophy, HDACs can modify the expression of critical metabolic enzymes; a recent review highlights the most relevant metabolic pathways regulated by HDACs [139]. For example, class II HDACs induce gluconeogenic enzyme G6Pase expression [108], while also triggering their other downstream target FoxO, a transcription factor associated with muscle protein degradation and autophagy [140], providing another link between epigenetic regulation and metabolism for muscle atrophy. Consistently, knocking out a facilitator of HDAC activity, NCoR1, increased muscle mass and promoted exercise capacity in mice [141]. As a consequence, HDAC inhibitors simultaneously act on protein and energy metabolism, potentially targeting distinct aspects of muscle wasting. As an example, butyrate, beyond interfering with myostatin-induced protein hypercatabolism, improves glucose metabolism in aged mice [125].

Considering epigenetics from a broader perspective, non-coding RNAs, especially miRNAs, participate in muscle mass regulation [142]. Changes in several micro-RNAs have been described in muscle atrophy conditions [143,144]. However, little focus has been afforded to investigate their role in mediating metabolic reactions. Although miRNAs have been suggested to function as biomarkers and potential therapeutic targets for cachexia and sarcopenia [145], to date, the only therapeutic study targeting skeletal muscle has been miR-23a/27a delivery, which preserved muscle mass in mice with diabetes-induced cachexia [146].

**Figure 4:**
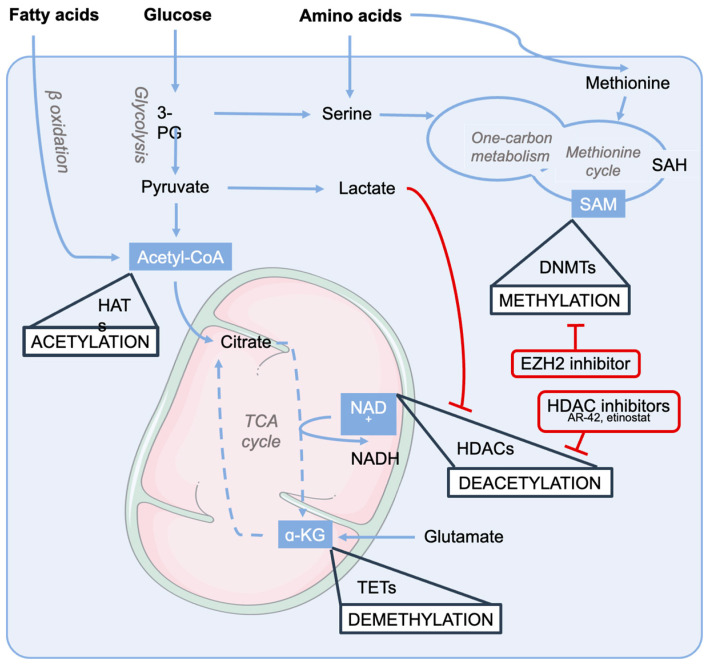
Simplified illustration of the connections between intermediate metabolites and epigenetic regulation. Epigenetic processes are written in upper case and highlighted with white boxes. Crucial metabolites for epigenetic modifications are highlighted with blue boxes. Metabolic pathways are written in italics. Abbreviations—3-PG: 3-phophoglyceric acid; Acetyl-CoA: acetyl coenzyme A; NAD^+^: nicotinamide adenine dinucleotide; NADH: nicotinamide adenine dinucleotide, reduced form; ɑ-KG: alpha ketoglutarate; TCA cycle: tricarboxylic acid cycle; HATs: histone acetyltransferases; HDACs: histone deacetylases; TETs: ten eleven translocases, i.e., ɑ-KG-dependent dioxygenases; SAM: S-adenosylmethionine; SAH: S-adenosylhomocysteine; DNMTs: DNA methyltransferases. Image created with PowerPoint and Smart Servier.

Overall, the importance of muscle metabolism in controlling muscle mass and function is growing and the specific pathways associated with the epigenetic status (Figure 4) are being elucidates; these factors could lead to the identification of prospective epigenetic drugs to counteract the metabolic dysfunction underlying sarcopenia and cachexia.

## 6. HDACs and BET Proteins in Skeletal Muscle Regeneration in Cachexia and Sarcopenia

HDACs can influence the differentiation of muscle precursor cells into mature muscle fibers. Altered HDAC activity might impede the regenerative potential of muscle tissue, contributing to the progression of sarcopenia. Impaired regeneration has been suggested as a potential factor contributing to the muscle loss observed in cancer cachexia. In fact, muscle tissues from tumor-bearing mice have been reported to show an accumulation of Pax7+ myogenic precursors that are unable to efficiently differentiate into myotubes [147,148]. Likewise, sarcopenic muscles experience a reduction in the number and function of muscle stem cells [73,149,150].

HDAC1 has been extensively shown to play a role in regulating myogenesis: TSA, VPA, and butyrate increase the differentiation potential of C2C12 myoblasts by promoting MyoD acetylation and modulating histone acetylation at specific gene promoters [150].

HDAC4, HDAC5, HDAC7, and HDAC9, which are categorized as class IIa HDACs, have been implicated in the regulation of myogenesis through their impact on myocyte enhancer factor 2 (MEF2) [151,152]. These HDACs physically interact with MEF2 [153] (Figure 3) and act to hinder the differentiation of myoblasts. Remarkably, the repression of MEF2 by class IIa HDACs occurs independently of their deacetylase activity, suggesting that the effects of HDACs are mediated by the recruitment of co-repressors or the exclusion of transcriptional activators [154,155].

Notably, both BRD3 and BRD4 play roles in regulating skeletal myogenesis. In differentiating C2C12 myoblasts, BRD4 knockdown is essential for myogenic differentiation, whereas downregulation of BRD3 enhances myogenic differentiation [156]. Roberts and colleagues demonstrated that BET inhibitor compounds hinder myogenic differentiation across various cell models. The inhibitory impact of pan-BET inhibitors on myogenesis was replicated by specifically depleting BRD4 through RNA interference and offered initial proof of the significant roles played by BET proteins, especially BRD4, in myogenic differentiation. This indicates the potential involvement of BRD4 in activating the muscle transcription program, potentially by binding to hyperacetylated pro-myogenic enhancers and/or promoters [156].

## 7. Conclusions

The current body of evidence, based on the initial use of HDAC and BET inhibitors in experimental models of sarcopenia and cachexia along with preliminary experiments conducted in models of muscle disuse and denervation, suggests that these two classes of inhibitors warrant further investigation to assess their full potential as a therapeutic avenue in the treatment of cachexia and sarcopenia. As newly developed inhibitors with improved safety profiles are tested, it is essential to comprehensively evaluate their long-term effects. Furthermore, it is relevant to assess the impact of second-generation BD-specific inhibitors in the context of cachexia and sarcopenia and determine which isoform-specific HDAC inhibitors are most effective in mitigating muscle wasting. Dual BET/HDAC inhibitor treatment may also have the potential to address multiple processes contributing to muscle loss simultaneously, keeping in mind that both cancer cachexia and aging sarcopenia are multifactorial and multiorgan syndromes that likely require the targeting of multiple pathways to obtain a clinically relevant phenotypical effect.

Furthermore, whereas a crosstalk between muscle and bone changes has been described both in cachexia [157,158] and sarcopenia [159,160], our understanding of how epigenetic factors influence the regulation of the muscle/bone interaction in muscle wasting remains quite limited.

HDAC inhibitors suppress osteoblastic progenitors and thus might not represent an effective approach for addressing conditions related to bone aging or cachexia, such as osteopenia and osteoporosis. In these conditions, maintaining a healthy reservoir of stem and progenitor cells is crucial for the generation of new bone tissue, and HDACs could potentially exacerbate the process of osteoclastogenesis. Current evidence hints at the possibility that selective inhibitors targeting HDAC6 [161] and activators of SIRTs could potentially stimulate bone formation or prevent bone loss [162]. However, further research is necessary to unravel the precise mechanisms underpinning these observed effects [163]. On the other hand, BET inhibitors have been shown to block osteoclastic activity and prevent the bone loss caused by ovariectomy (OVX) in vivo [164], suggesting that the pharmacological blockade of BET proteins may represent a therapeutic avenue for osteoporosis.

At the molecular level, a more precise evaluation of the genome-wide distribution of HDACs and BET proteins in skeletal muscle during cachexia and sarcopenia will enable us to uncover novel regulatory circuits and gene categories that are directly regulated by these chromatin factors. This insight will help us better understand the modulation of these gene categories following treatments with epigenetic inhibitors. Furthermore, a more in-depth exploration of the interaction between HDACs or BET proteins and acetylated proteins that play a crucial role in muscle wasting, such as FoxO, p65/NF-κB, STAT1/3, and PGC-1α, may reveal novel regulatory dynamics mediated by the interaction of acetylated transcription factors and BET bromodomains, extending their relevance beyond histones.

## Figures and Tables

**Table 1:** HDAC and BET inhibitors and their molecular targets.

Group	Compound	Molecule Target
HDAC inhibtor (hydroxamic acids)	Vorinostat (SAHA)	Class I, Class II, Class IV
	Trichostatin A (TSA)	Class I, Class II, Class IV
	Belinostat (PXD101)	Class I, Class II, Class IV
	Givinostat (ITF2357)	Class I, Class II
	AR-42	Class I, Class IIb
	Panobinostat (LBH689)	Class I, Class II, Class IV
HDAC inhibtor (benzamides)	Entinostat (MS-275)	Class I
	Mocetinostat	Class I and Class IV
	Chidamide	Class I HDAC1, HDAC2, HDAC3, and Classe IIb HDAC10
	Tacedinaline	Class I
	Domatinostat	Class I
	Zabadinostat (CXD101)	HDAC1, HDAC2, and HDAC3
HDAC inhibtor (cyclic peptide)	Romidepsin	Class I
HDAC inhibtor (short chain fatty acid)	Valproic acid (VPA)	Class I, Class IIa
	Butyrate	Class I, Class IIa
HDAC inhibtor	CKD-504	HDAC6
	CKD-506	HDAC6
	CS3003	HDAC6
	HG116KA2507	HDAC6
BET inhibitor	JQ1	BET proteins
	PFI-1	BET proteins
	I-BET762	BET proteins
	OTX015	BET proteins
	CPI-0610	BET proteins
	RVX-208	BD2
	GSK778	BD1
